# A genome-wide association study identifies a novel locus on chromosome 18q12.2 influencing white cell telomere length

**DOI:** 10.1136/jmg.2008.064956

**Published:** 2009-04-24

**Authors:** M Mangino, J B Richards, N Soranzo, G Zhai, A Aviv, A M Valdes, N J Samani, P Deloukas, T D Spector

**Affiliations:** 1 Department of Twin Research and Genetic Epidemiology, King’s College London, London, UK; 2 Department of Medicine, Jewish General Hospital, Faculty of Medicine, McGill University, Montréal, Québec, Canada; 3 Wellcome Trust Sanger Institute, Wellcome Trust Genome Campus, Hinxton, UK; 4 Centre of Human Development and Aging, New Jersey Medical School, Newark, New Jersey, USA; 5 Department of Cardiovascular Sciences, University of Leicester, Leicester, UK

## Abstract

**Background:**

Telomere length is a predictor for a number of common age related diseases and is a heritable trait.

**Methods and results:**

To identify new loci associated with mean leukocyte telomere length we conducted a genome wide association study of 314 075 single nucleotide polymorphisms (SNPs) and validated the results in a second cohort (n for both cohorts combined  =  2790). We identified two novel associated variants (rs2162440, p = 2.6×10^−6^; and rs7235755, p = 5.5×10^−6^) on chromosome 18q12.2 in the same region as the *VPS34/PIKC3C* gene, which has been directly implicated in the pathway controlling telomere length variation in yeast.

**Conclusion:**

These results provide new insights into the pathways regulating telomere homeostasis in humans.

Telomeres are nucleoprotein structures capping and protecting the ends of chromosomes. Because of the “end replication problem”,^1^ telomeres shorten with each cell division and leucocyte telomere length has been shown to decrease with age at a rate of 20–40 base pairs per year.^2^ ^3^ Telomere attrition is enhanced by inflammation and oxidative stress and short telomere length is an independent predictor of age related diseases such as hypertension, myocardial infarction, congestive heart failure, vascular dementia, osteoporosis, osteoarthritis and Alzheimer’s disease.^3^

There is wide inter-individual variability in telomere length at birth and at subsequent ages. Both twin studies and intra-familial correlation analysis have identified a genetic influence (from 40% to 80%) on telomere length variation.^4^ ^5^ Genome-wide linkage studies have mapped QTLs for this trait to chromosomes 12q12.22^5^ and 14q23.2.^4^ More recently Mangino *et al*^6^ refined the chromosome 12q12.22 locus and described an associated polymorphism (rs2630778) in the *BICD1* gene. To date, none of these findings have been replicated, possibly due to difficulties in measuring this trait in a large number of samples and due to lack of high correlation between the methods used to measure telomere length.

Genome-wide association (GWA) analysis is a powerful tool for unlocking the genetic basis of complex traits and has recently provided novel insights into the genetic architecture of many common diseases and traits.^7^ ^8^ We therefore undertook a GWA scan to identify common alleles that may influence telomere length. Our findings indicate that single nucleotide polymorphisms (SNPs) rs2162440 and rs7235755 on chromosome 18q12.2 are associated with short telomere length in two independent datasets of European descent.

## METHODS

We conducted a two stages GWA study on 2790 individuals from the UK Adult Twin Register (table 1), in which we evaluated 314 075 SNPs. The design and methodology of the GWA study is described in detail elsewhere.^7^ In brief, the discovery sample consisted of 1625 women from the St Thomas’ UK Adult Twin Registry,^9^ a large cohort of twins historically developed to study the heritability and genetics of diseases with a higher prevalence among women. The sample is not enriched for any particular disease or trait and is representative of the British general population.^4^ The replication cohort included 1165 subjects of both genders (table 1) from the UK Twin Registry who were unrelated to the individuals from the discovery sample.

**Table 1:** Characteristics of the 2790 individuals assessed for telomere length variation

	Twins UK discovery cohort	Replication cohort	Total sample
Subjects assessed for TRF	1625	1165	2790
Age (years)*	47.9 (12.6)	49.2 (13.6)	48.5 (13.1)
Males*	–	48.1 (13.8)	48.1 (13.8)
Females*	47.9 (12.6)	49.5 (13.5)	48.5 (13.0)
Sex	–	264	264
Males	1625	901	2526
Females			
LTL*	7.02 (0.67)	6.91 (0.68)	6.97 (0.68)
Males*	–	6.68 (0.69)	6.68 (0.69)
Females*	7.02 (0.67)	6.98 (0.66)	7.01 (0.67)

LTL, leucocyte telomere length.

*Values presented as mean (SD).

Leucocyte telomere length (LTL) was derived by using Southern blot analysis in duplicate to measure the mean terminal restriction fragment.^10^ The coefficient of variation for this measurement was 1.5%. Because all the individuals of the discovery cohort were females, telomere length was only adjusted for age. After adjustment, the trait was normally distributed in the sample.

Genomic DNA was subjected to SNP genotyping via the Infinium assay (Illumina, San Diego, California, USA), using three fully compatible BeadChip microarrays (HumanHap300-Duo, HumanHap300 and HumanHap550), according to the manufacturer’s protocols.

We excluded 733 SNPs that had a low call rate (⩽90%), 2704 SNPs that had Hardy–Weinberg p values <10^−4^, and 725 SNPs with minor allele frequencies <1%. We also removed subjects where genotyping failed for >2% of SNPs. We retained for the analysis 98.7% (314 075) of all available SNPs. Statistical analysis was carried out with MERLIN (version 1.1.2)^11^ using the score test (—fastAssoc), while accounting for family structure and twin zygosity.^12^

## RESULTS

In the discovery sample (n = 1625) the strongest association was recorded for rs7374458 on chromosome 3 (5.20×10^−6^). We also identified 28 SNPs with a p value of ⩽10^−4^ and 316 SNPs with a p value of ⩽10^−3^. We visually inspected all the signal intensity plots of these SNPs and excluded the markers that had been miscalled (11.3%).

Since none observed p values reached a genome-wide significance level after correcting for multiple testing, we adopted the conservative approach of selecting for replication only those polymorphisms with a p value <10^−3^ that were ≈100 Kb from other associated SNPs (p⩽1.0×10^−2^). Following these criteria, we identified 15 associated loci including a total of 41 SNPs with the p values for the lead SNPs ranging from 5.20×10^−6^ to 9.7×10^−4^ (table 2).

**Table 2:** Summary of the 15 loci analysed in discovery and replication cohorts

Chromosome	Marker	Position	Allele	β GWA (SE)	p Values GWA	β replication (SE)	p Values replication	β combined (SE)	p Values combined	Locus
1p35.2	rs7514514	31180643	G	−0.109 (0.025)	1.30E−05	0.035 (0.030)	3.30E−01	−0.049 (0.019)	1.45E−02	PUM1*
1p35.2	rs12406355	31190045	A	0.089 (0.024)	1.60E−04	0 (0.027)	1.00E+00	0.05 (0.018)	6.30E−03	PUM1*
1q42.13	rs238102	227549771	C	0.08 (0.025)	9.73E−05	0.027 (0.030)	3.80E−01	0.059 (0.019)	2.10E−03	C1orf96§
1q42.13	rs238099	227575528	A	0.068 (0.023)	2.58E−04	0.006 (0.030)	8.60E−01	0.045 (0.018)	1.70E−02	C1orf96§
1q42.13	rs7549589	227586550	A	0.062 (0.024)	1.38E−03	−0.002 (0.034)	9.70E−01	0.037 (0.018)	5.70E−02	C1orf96§
1q44	rs3102458	242606491	A	0.086 (0.028)	2.10E−03	−0.002 (0.033)	1.00E+00	0.045 (0.022)	2.40E−02	C1orf100*
1q44	rs3123710	242611441	A	−0.076 (0.023)	1.01E−03	0.015 (0.028)	4.20E−01	−0.041 (0.018)	3.30E−02	C1orf100*
1q44	rs3003211	242679607	A	−0.082 (0.025)	8.40E−04	0.018 (0.030)	4.70E−01	−0.042 (0.019)	3.30E−02	ADSS*
2q22.2	rs1376749	143683862	G	−0.123 (0.032)	1.10E−04	−0.059 (0.040)	1.49E−01	−0.102 (0.025)	6.20E−05	ARHGAP15*
2q22.2	rs12993643	143749432	G	−0.093 (0.025)	1.80E−04	−0.013 (0.030)	6.60E−01	−0.062 (0.019)	1.32E−03	ARHGAP15*
2q22.2	rs4662198	143764197	C	−0.095 (0.028)	6.00E−04	−0.025 (0.035)	4.70E−01	−0.069 (0.022)	1.50E−03	ARHGAP15*
2q33.1	rs1036533	201105969	G	−0.157 (0.044)	3.90E−04	−0.048 (0.058)	6.10E−01	−0.115 (0.035)	8.90E−04	SGOL2†
2q33.1	rs10497853	201183402	A	−0.16 (0.045)	4.30E−04	−0.066 (0.057)	2.50E−01	−0.125 (0.036)	1.03E−03	AOX1*
3p22–p21.3	rs1858740	38413376	G	−0.081 (0.023)	3.40E−04	0.009 (0.028)	8.30E−01	−0.045 (0.018)	1.34E−02	XYLB*
3p22–p21.3	rs4407366	38496429	A	−0.103 (0.023)	8.80E−06	0.04 (0.033)	2.60E−01	−0.054 (0.019)	6.50E−03	ACVR2B*
3p22–p21.3	rs7374458	38506215	C	−0.105 (0.023)	5.20E−06	0.02 (0.028)	4.80E−01	−0.054 (0.018)	4.00E−03	ACVR2B‡
3p14.3	rs3774601	53797933	G	−0.1 (0.024)	3.10E−05	0.049 (0.028)	8.60E−02	−0.038 (0.019)	3.80E−02	CACNA1D*
3p14.3	rs3774605	53805807	G	−0.1 (0.024)	3.70E−05	0.053 (0.028)	6.00E−02	−0.035 (0.018)	5.70E−02	CACNA1D*
3p14.3	rs3774609	53807943	A	−0.092 (0.024)	1.20E−04	0.04 (0.028)	1.48E−01	−0.036 (0.023)	7.40E−02	CACNA1D*
4q31.21	rs1907107	143449084	A	0.073 (0.023)	3.40E−04	−0.009 (0.026)	7.40E−01	0.034 (0.018)	4.90E−02	INPP4B*
4q31.21	rs2635429	143463006	G	0.067 (0.023)	2.72E−04	−0.02 (0.027)	4.80E−01	0.029 (0.018)	1.01E−01	INPP4B*
4q31.21	rs1497393	143493161	G	−0.066 (0.023)	1.48E−04	0.025 (0.028)	3.70E−01	−0.029 (0.018)	1.10E−01	INPP4B*
8p23.1	rs4841067	8784655	A	0.139 (0.032)	1.90E−05	0.01 (0.038)	8.30E−01	0.088 (0.025)	5.70E−04	MFHAS1*
8p23.1	rs11778913	8852300	A	0.069 (0.026)	8.10E−03	0.057 (0.033)	8.40E−02	0.067 (0.021)	1.48E−03	THEX1§
8p23.1	rs11249943	9645273	A	−0.084 (0.029)	3.60E−03	0.021 (0.033)	5.20E−01	−0.037 (0.022)	7.80E−02	TNKS*
8p23.1	rs6989782	9647948	G	−0.076 (0.025)	2.52E−04	−0.003 (0.030)	8.80E−01	−0.048 (0.019)	1.14E−02	TNKS*
9q31.2	rs1570504	110094630	C	−0.107 (0.033)	1.24E−03	0.016 (0.031)	4.60E−01	−0.055 (0.026)	2.90E−02	ACTL7B‡
9q31.2	rs1535619	110094886	A	−0.075 (0.031)	1.50E−02	0.004 (0.037)	9.10E−01	−0.04 (0.024)	8.30E−02	ACTL7B‡
9q31.2	rs7028041	110096538	G	−0.109 (0.034)	1.23E−03	0.017 (0.023)	5.50E−01	−0.05 (0.026)	4.40E−02	ACTL7B‡
13q31.3	rs9301921	93432990	A	0.08 (0.031)	5.25E−04	0.059 (0.037)	1.06E−01	0.074 (0.024)	2.20E−03	GPC6*
13q31.3	rs1415736	93444937	G	0.081 (0.031)	3.70E−04	0.061 (0.043)	1.60E−01	0.075 (0.026)	3.50E−03	GPC6*
14q22.2	rs4898848	54266336	A	0.072 (0.024)	4.53E−04	−0.001 (0.028)	8.00E−01	0.04 (0.018)	2.90E−02	SAMD4*
14q22.2	rs6572971	54280838	G	0.1 (0.032)	7.90E−04	−0.021 (0.039)	6.00E−01	0.052 (0.025)	4.80E−02	SAMD4*
14q22.2	rs1957356	54288744	G	0.092 (0.029)	9.65E−05	0.03 (0.033)	3.90E−01	0.064 (0.022)	2.70E−03	SAMD4*
16q24.2–q24.3	rs17214677	82008463	A	0.111 (0.037)	2.80E−03	−0.074 (0.046)	1.12E−01	0.038 (0.029)	2.10E−01	CDH13*
16q24.2–q24.3	rs11861722	82125547	A	0.124 (0.038)	9.70E−04	−0.098 (0.043)	2.30E−02	0.021 (0.029)	4.20E−01	CDH13*
16q24.2–q24.3	rs9934005	82137699	G	0.088 (0.027)	9.20E−04	−0.028 (0.031)	3.80E−01	0.034 (0.020)	8.50E−02	CDH13*
16q24.2–q24.3	rs12598842	82145455	A	−0.059 (0.023)	8.80E−03	−0.007 (0.023)	1.00E+00	−0.038 (0.018)	4.40E−02	CDH13*
18q12.2	**rs2162440**	33468004	G	−0.104 (0.029)	**2.50E**−**04**	−0.119 (0.035)	**1.08E**−**03**	−0.106 (0.022)	**2.60E**−**06**	BRUNOL4§
18q12.2	**rs7235755**	33470259	G	−0.104 (0.028)	**2.60E**−**04**	−0.114 (0.035)	**2.00E**−**03**	−0.103 (0.022)	**5.50E**−**06**	BRUNOL4§
18q12.2	rs2217127	33475628	C	−0.08 (0.025)	1.30E−03	−0.047 (0.035)	2.20E−01	−0.063 (0.020)	2.10E−03	BRUNOL4§

GWA, genome wide association; SE, standard error.

*Intron; †coding; ‡flanking 3′ UTR; §flanking 5′UTR.

Values in bold represent the two associated variants.

These 41 selected SNPs were genotyped in the replication cohort (n = 1165) using Sequenom iPLEX (San Diego, California, USA) technology. Because the replication cohort included both males and females, LTL values were adjusted for both gender and age. After adjustment the trait was again normally distributed. To control for multiple testing, we used an SNP spectral decomposition method proposed by Nyholt^13^ and modified by Li and Ji.^14^ After spectral decomposition of the linkage disequilibrium (LD) matrices of the 41 analysed SNPs, the corrected threshold of statistical significance in the replication stage was estimated at p⩽2.1×10^−3^ which is a conservative correction for the number of independent SNPs tested in the replication sample. The results of the association analysis are reported in table 2 and show that we were able to replicate the association observed in the GWA sample for two markers, rs2162440 and rs7235755, both mapping to a 2.2 Kb region of chromosome 18q12.2.

Since the discovery cohort included only females, we also performed a gender specific analysis on the replication population in order to test if the genetic variants may be associated with telomere lengths only for females. The result showed that for both SNPs the direction of the trend was consistent between genders in the replication cohort (rs2162440: −100 (44) base pairs (bp) for females and −140 (70) bp for males; rs7235755 −94 (42) bp for females and −138 (71) bp for males) and between females of the two cohorts (rs2162440: −104 (29) bp for female in discovery and −100 (44) bp for females in replication; rs7235755 −104 (28) bp for female in discovery and −94 (42) bp for females in replication). Although borderline (due to small sample size), p values were statistically significant for both SNPs in both genders in the replication cohort (rs2162440: females p = 0.012, males p = 0.046; rs7235755: females p = 0.02, males p = 0.049).

The joint analysis of genotyped data from the two cohorts yielded combined p values of 2.60×10^−6^ (rs2162440) and 5.50×10^−6^ (rs7235755). Our analysis also indicated that the G alleles of both SNPs were associated with shorter telomeres (−106 (22) bp for rs2162440 and −103 (22) bp for rs7235755), extrapolating to an approximate 5 years of telomere erosion based on estimates of loss with age.

## DISCUSSION

Although our results are unlikely to be artefacts because the identified SNPs were replicated in two independent cohorts, we do believe that our power for identifying association was reduced by the known limitations of the measurement technique.^15^ Therefore, we can only detect common variants. Indeed, it is likely that there are more loci with small genetic effect that we did not detect because of the stringent thresholds for statistical significance employed in this study. This would explain why we did not detect loci such as those previously identified on chromosome 12q12.22 and 14q23.2.

According to NCBI build 36, the associated polymorphisms map to a 48 Kb LD block within a gene desert, between the Bruno-like 4 (*BRUNOL4*, NM_020180) and *VPS34* (also known as *PIK3C3*, NM_002647) genes. The identified SNPs (or another variant present in the LD block) might be influencing the expression of either transcript through long range control, as has been demonstrated for other genes.^16^ This hypothesis is supported by the observation that the associated 48 Kb LD block lies in a highly conserved genomic segment. The two associated variants map ∼70 Kb away from *BRUNOL4* and 4.3 Mb away from *VPS34*. *BRUNOL4* is a member of the CELF/Bruno-like family, which encodes proteins bearing highly conserved RNA recognition motif. RNA binding proteins are important elements that control normal cell functions, regulating events such as RNA processing, mRNA transport, stability and translation. *VPS34* is a component of the phosphoinositide (PI) 3 kinase family which includes proteins that regulate several aspect of the cell physiology.^17^ Interestingly, *VPS34* yeast orthologue (Vps34) has been directly involved in the pathway which regulates telomere length variation.^18^

In conclusion, we provide evidence from two independent cohorts for a new locus on chromosome 18q12.2 associated with short telomere length in humans. These data provide new insights into the likely pathways and mechanisms regulating telomere length in humans.
